# Television Viewing and Incident Cardiovascular Disease: Prospective Associations and Mediation Analysis in the EPIC Norfolk Study

**DOI:** 10.1371/journal.pone.0020058

**Published:** 2011-05-25

**Authors:** Katrien Wijndaele, Søren Brage, Hervé Besson, Kay-Tee Khaw, Stephen J. Sharp, Robert Luben, Amit Bhaniani, Nicholas J. Wareham, Ulf Ekelund

**Affiliations:** 1 Medical Research Council Epidemiology Unit, Cambridge, United Kingdom; 2 Research Foundation Flanders, Brussels, Belgium; 3 Department of Movement and Sports Sciences, Ghent University, Ghent, Belgium; 4 Department of Public Health and Primary Care, Institute of Public Health, University of Cambridge, Cambridge, United Kingdom; Innsbruck Medical University, Austria

## Abstract

**Background:**

Although television viewing time is detrimentally associated with intermediate cardiovascular risk factors, the relationship with incident total (i.e. combined fatal and non-fatal) cardiovascular disease (CVD), non-fatal CVD and coronary heart disease is largely unknown. This study examined whether television viewing time is associated with these three outcomes, independently of physical activity energy expenditure and other confounding variables.

**Methodology/Principal Findings:**

A population-based cohort of 12,608 men and women (aged 61.4±9.0), free from stroke, myocardial infarction and cancer at baseline in 1998–2000 were followed up until 2007 (6.9±1.9 years). Participants self-reported education, smoking, alcohol use, antihypertensive, lipid lowering and antidepressant medication, disease history, total energy intake, sleep duration, physical activity and television viewing. BMI, waist circumference, blood pressure, triglycerides, HDL cholesterol and glycated haemoglobin (HbA_1c_) were measured by standardized procedures; a clustered metabolic risk score was constructed. Every one hour/day increase in television viewing was associated with an increased hazard for total (HR = 1.06, 95%CI = 1.03–1.08; 2,620 cases), non-fatal CVD (HR = 1.06, 95%CI = 1.03–1.09; 2,134 cases), and coronary heart disease (HR = 1.08, 95%CI = 1.03–1.13; 940 cases), independent of gender, age, education, smoking, alcohol, medication, diabetes status, CVD family history, sleep duration and physical activity energy expenditure. Energy intake, BMI, waist circumference, blood pressure, triglycerides, HDL cholesterol, HbA_1c_ and the clustered metabolic risk score only partially mediated these associations.

**Conclusions:**

These results indicate that the most prevalent leisure time (sedentary) behaviour, television viewing, independently contributes to increased CVD risk. Recommendations on reducing television viewing time should be considered.

## Introduction

Sedentary behaviour (i.e. excessive sitting, as distinct from insufficient physical activity), occupies a substantial part of the waking day in modern society (e.g. 55% in the US) [1]. Increasing evidence from prospective studies indicates detrimental associations between excessive sitting, and television viewing time in particular, and intermediate cardiovascular risk factors, which are independent of physical activity and other relevant covariates. These include weight gain and incident obesity [2]–[4], dyslipidaemia [5], hypertension [6] and insulin resistance and diabetes [3], [7], [8]. Potential mechanistic explanations for these associations are beginning to be unravelled [9].

Few prospective studies however have so far examined independent sedentary behaviour effects on more downstream incident cardiovascular disease (CVD). Excessive total or non-occupational sitting time increased cardiovascular mortality hazard in Canadian [10] and middle-aged US adults [11] and total CVD hazard in US post-menopausal women [12] following adjustment for leisure time physical activity. Specifically for television viewing, an increased hazard of prolonged viewing time on cardiovascular mortality was found in Australian [13] and UK adults [14], independent of leisure time exercise and total physical activity respectively, but not in US men from the Aerobics Center Longitudinal Study [15]. One recent study in Scottish adults examined the prospective associations between leisure screen time and total (i.e. combined fatal and non-fatal) incident CVD [16]. However, so far it has not been studied yet whether television viewing is independently associated with incident coronary heart disease or other specific types of CVD, such as cardiac failure and stroke. Compared to previous studies focusing on cardiovascular mortality [13]–[15], examining the relationship between television viewing and non-fatal CVD is of additional value, as morbidity and incapacity caused by non-fatal cardiovascular events are major contributors to the economic burden for society and the health care system [17], [18]. In addition, the first CVD event is most often more proximal in the cardiovascular disease process to the exposure of interest, and so may provide a more complete picture of the potential public health hazard associated with television viewing in terms of incident CVD.

Similar to the US and Australia, television viewing is the predominant leisure time sedentary behaviour in British adults and one of the three main activities besides sleeping and working [19]–[21]. Therefore it is likely to be representative of total leisure time spent sitting [22]. Further, it might be more susceptible to voluntary change compared to other sources of excessive sitting, such as occupational sitting. Moreover, compared to sitting in general, television viewing is associated with additional factors potentially mediating associations with CVD, such as increased energy intake by exposure to unhealthy food advertisements [23]. This may result in detrimental associations which differ in effect size from those caused by sitting per se.

In the EPIC Norfolk prospective population study, we examined the independent association between television viewing time and incident total cardiovascular, non-fatal cardiovascular and coronary heart disease events in a population of healthy middle-aged white adults. We determined whether any such associations were independent of total (at home, to work, at work and during leisure-time) physical activity energy expenditure (PAEE) and other relevant confounders. Furthermore, we also examined to what extent these associations were mediated by a range of intermediate cardiovascular risk factors.

## Results

### Descriptive characteristics

During 87,572 person-years of follow-up (men: 36,846, women: 50,726), 2,620 participants developed incident CVD (1,351 men, 1,269 women). Out of those 2,620 incident CVD cases, 2,134 (81.5%) were non-fatal CVD events (men: 1,064, women: 1,070) and 940 (35.9%) were (fatal or non-fatal) coronary heart disease events (men: 625, women: 315). As shown in Table 1, participants who developed CVD watched television at baseline for on average half an hour/day more than those who did not (any and non-fatal CVD: 0.4, coronary heart disease: 0.5 hours/day). Comparing baseline characteristics by tertiles of the exposure of interest (see Table 2), higher levels of television were associated with gradually less favourable profiles of most covariates in men and women. Television viewing (hours/day) and PAEE (MET*hours/week) were weakly inversely correlated (Spearman correlation: −0.17, *P*<0.01).

**Table 1:** Descriptive characteristics (mean (SD) or No. (%)) at baseline between incident cardiovascular event cases and non-cases in 12,608 men and women in EPIC Norfolk, 1998–2007.

	Any cardiovascular event		Non-fatal cardiovascular event		Coronary heart disease event	
Characteristics	Incident casesn = 2,620	Non-casesn = 9,988	Incident casesn = 2,134	Non-casesn = 10,474	Incident casesn = 940	Non-casesn = 11,668
Follow-up time (yrs)	4.3 (2.2)	7.6 (1.0)*	4.4 (2.2)	7.5 (1.3)*	4.3 (2.2)	7.6 (1.1)*
Male gender, No. (%)	1,351 (51.6)	4,114 (41.2)*	1,064 (49.9)	4,401 (42.0)*	625 (66.5)	4,840 (41.5)*
Age (yrs)	66.7 (8.1)	60.0 (8.7)*	65.9 (8.1)	60.4 (8.9)*	67.7 (7.8)	60.8 (8.9)*
Education level, No. (%)						
Low	1,055 (40.3)	2,988 (29.9)*	846 (39.6)	3,197 (30.5)*	378 (40.2)	3,665 (31.4)*
O level	252 (9.6)	1,150 (11.5)	210 (9.8)	1,192 (11.4)	87 (9.3)	1,315 (11.3)
A level	1,046 (39.9)	4,265 (42.7)	850 (39.8)	4,461 (42.6)	384 (40.9)	4,927 (42.2)
Degree	267 (10.2)	1,585 (15.9)	228 (10.8)	1,624 (15.5)	91 (9.7)	1,761 (15.1)
Cigarette smoking, No. (%)						
Current	243 (9.3)	790 (7.9)*	176 (8.2)	857 (8.2)*	106 (11.3)	927 (7.9)*
Former	1,259 (48.1)	3,974 (39.8)	1,014 (47.5)	4,219 (40.3)	499 (53.1)	4,734 (40.6)
Never	1,118 (42.6)	5,224 (52.3)	944 (44.2)	5,398 (51.5)	335 (35.6)	6,007 (51.5)
Alcohol consumption (units/wk)	6.7 (9.4)	7.0 (9.1)	6.7 (9.3)	7.0 (9.1)	6.9 (9.9)	7.0 (9.1)
Antihypertensive medication, No. (%)	1,062 (40.5)	1,366 (13.7)*	859 (40.3)	1,569 (15.0)*	374 (39.8)	2,054 (17.6)*
Lipid lowering medication, No. (%)	134 (5.1)	240 (2.4)*	113 (5.3)	261 (2.5)*	72 (7.7)	302 (2.6)*
Antidepressant medication, No. (%)	203 (7.7)	579 (5.8)*	161 (7.5)	621 (5.9)‡	67 (7.1)	715 (6.1)
Baseline diabetes, No. (%)	138 (5.3)	203 (2.0)*	109 (5.1)	232 (2.2)*	57 (6.1)	284 (2.4)*
Family history of CVD, No. (%)	1,439 (54.9)	4,983 (49.9)*	1,175 (55.1)	5,247 (50.1)*	542 (57.7)	5,880 (50.4)*
Total energy intake (kj/day)	8,355.9 (2,399.8)	8,208.5 (2,382.8)†	8,329.7 (2,386.6)	8,220.3 (2,386.8)	8,462.9 (2,456.1)	8,220.9 (2,380.6)‡
Fruit and vegetable intake (g/day)	476.1 (241.4)	475.9 (248.5)	480.6 (241.3)	475.0 (248.2)	448.1 (225.0)	478.2 (248.6)‡
Saturated fatty acids intake (%)	12.8 (3.5)	12.6 (3.4)†	12.7 (3.4)	12.7 (3.4)	12.9 (3.5)	12.7 (3.4)†
Sleep duration (hrs/day)	8.6 (0.9)	8.5 (0.8)*	8.6 (0.9)	8.5 (0.8)*	8.6 (0.9)	8.5 (0.8)*
Body mass index (kg/m^2^)	27.4 (4.1)	26.4 (3.9)*	27.5 (4.1)	26.4 (3.9)*	27.3 (3.9)	26.6 (3.9)*
Waist circumference (cm)	91.7 (12.2)	86.8 (12.3)*	91.5 (12.0)	87.1 (12.4)*	93.6 (11.8)	87.3 (12.4)*
Triglycerides (mmol/L)	2.06 (1.16)	1.81 (1.05)*	2.06 (1.18)	1.82 (1.05)*	2.19 (1.23)	1.83 (1.06)*
HDL cholesterol (mmol/L)	1.41 (0.45)	1.52 (0.46)*	1.41 (0.45)	1.51 (0.46)*	1.31 (0.41)	1.51 (0.46)*
Systolic blood pressure (mm Hg)	143.7 (18.5)	132.5 (17.2)*	143.2 (18.4)	133.1 (17.5)*	142.1 (18.7)	134.2 (17.9)*
Diastolic blood pressure (mm Hg)	85.8 (15.0)	81.2 (10.8)*	85.8 (15.6)	81.4 (10.9)*	84.2 (12.3)	82.0 (11.9)*
HbA_1c_ concentration (%)	5.64 (0.82)	5.43 (0.58)*	5.60 (0.78)	5.45 (0.60)*	5.71 (0.84)	5.46 (0.62)*
Clustered metabolic risk score	0.21 (0.60)	−0.09 (0.58)*	0.20 (0.60)	−0.08 (0.59)*	0.23 (0.59)	−0.05 (0.59)*
Physical activity (MET*hrs/day)	14.5 (7.5)	17.6 (7.9)*	14.9 (7.6)	17.4 (7.9)*	13.4 (7.2)	17.3 (7.9)*
TV viewing time (hrs/day)	3.4 (1.5)	3.0 (1.4)*	3.4 (1.5)	3.0 (1.4)*	3.5 (1.5)	3.0 (1.4)*

*P<0.001;

†*P*<0.05;

‡*P*<0.01 between incident cardiovascular event cases and non-cases;

CVD: cardiovascular disease.

**Table 2:** Descriptive characteristics (mean (SD) or No. (%)) at baseline by television viewing tertiles (lowest: <2.5, middle: 2.5–3.6, highest: >3.6 hours/day) in 12,608 men and women in EPIC Norfolk.

	Television viewing tertiles		
Characteristics	Lowest	Middle	Highest
**Men** (n)	1,947	1,859	1,659
Age (yrs)	59.8 (8.6)	61.7 (9.1)	64.9 (8.6)*
Education level, No. (%)			
Low	339 (17.4)	479 (25.8)	611 (36.8)*
O level	161 (8.3)	173 (9.4)	164 (9.9)
A level	909 (46.7)	951 (51.1)	748 (45.1)
Degree	538 (27.6)	256 (13.7)	136 (8.2)
Cigarette smoking, No. (%)			
Current	130 (6.7)	147 (7.9)	169 (10.2)*
Former	947 (48.6)	1,008 (54.2)	980 (59.1)
Never	870 (44.7)	704 (37.9)	510 (30.7)
Alcohol consumption (units/wk)	10.9 (11.9)	9.6 (11.1)	9.5 (11.8)*
Antihypertensive medication, No. (%)	279 (14.3)	340 (18.3)	394 (23.7)*
Lipid lowering medication, No. (%)	46 (2.4)	51 (2.7)	60 (3.6)
Antidepressant medication, No. (%)	72 (3.7)	70 (3.8)	84 (5.1)
Baseline diabetes, No. (%)	50 (2.6)	67 (3.6)	69 (4.2)†
Family history of CVD, No. (%)	942 (48.4)	915 (49.2)	839 (50.6)
Total energy intake (kj/day)	8,874.6 (2,546.4)	8,933.4 (2,483.4)	8,889.3 (2,508.2)
Fruit and vegetable intake (g/day)	442.1 (226.9)	420.9 (204.9)	413.1 (236.1)‡
Saturated fatty acids intake (%)	13.0 (3.3)	13.1 (3.3)	13.2 (3.3)
Sleep duration (hrs/day)	8.3 (0.8)	8.3 (0.9)	8.4 (0.9)*
Body mass index (kg/m^2^)	26.3 (3.2)	26.8 (3.3)	27.3 (3.4)*
Waist circumference (cm)	94.1 (9.5)	95.8 (9.5)	97.8 (9.8)*
Triglycerides (mmol/L)	1.97 (1.12)	2.06 (1.11)	2.24 (1.36)*
HDL cholesterol (mmol/L)	1.35 (0.39)	1.28 (0.35)	1.25 (0.37)*
Systolic blood pressure (mm Hg)	134.0 (17.1)	137.4 (17.3)	140.2 (17.4)*
Diastolic blood pressure (mm Hg)	83.0 (10.8)	84.5 (10.9)	86.0 (16.2)*
HbA_1c_ concentration (%)	5.44 (0.56)	5.49 (0.70)	5.57 (0.76)*
Clustered metabolic risk score	−0.15 (0.57)	−0.02 (0.55)	0.11 (0.57)*
Physical activity (MET*hrs/day)	18.5 (9.1)	17.9 (9.3)	14.5 (8.2)*
**Women** (n)	2,299	2,407	2,437
Age (yrs)	58.4 (8.9)	60.7 (8.6)	63.5 (8.5)*
Education level, No. (%)			
Low	505 (22.0)	871 (36.2)	1,238 (50.8)*
O level	280 (12.2)	321 (13.3)	303 (12.4)
A level	956 (41.6)	960 (39.9)	787 (32.3)
Degree	558 (24.2)	255 (10.6)	109 (4.5)
Cigarette smoking, No. (%)			
Current	169 (7.4)	186 (7.7)	232 (9.5)*
Former	694 (30.2)	784 (32.6)	820 (33.6)
Never	1,436 (62.4)	1,437 (59.7)	1,385 (56.9)
Alcohol consumption (units/wk)	5.2 (6.1)	4.7 (5.6)	3.9 (5.2)*
Antihypertensive medication, No. (%)	360 (15.7)	459 (19.1)	596 (24.5)*
Lipid lowering medication, No. (%)	40 (1.7)	76 (3.2)	101 (4.1)*
Antidepressant medication, No. (%)	156 (6.8)	178 (7.4)	222 (9.1)‡
Baseline diabetes, No. (%)	31 (1.3)	51 (2.1)	73 (3.0)*
Family history of CVD, No. (%)	1,197 (52.1)	1,272 (52.8)	1,257 (51.6)
Total energy intake (kj/day)	7,620.7 (2,088.9)	7,661.3 (2,154.6)	7,966.2 (2,229.7)*
Fruit and vegetable intake (g/day)	528.3 (260.6)	513.0 (255.3)	498.2 (255.2)‡
Saturated fatty acids intake (%)	12.3 (3.5)	12.3 (3.4)	12.5 (3.4)†
Sleep duration (hrs/day)	8.5 (0.8)	8.6 (0.8)	8.7 (0.8)*
Body mass index (kg/m^2^)	25.7 (4.1)	26.5 (4.3)	27.2 (4.5)*
Waist circumference (cm)	79.6 (10.1)	81.5 (10.6)	83.9 (10.9)*
Triglycerides (mmol/L)	1.53 (0.88)	1.68 (0.95)	1.84 (0.97)*
HDL cholesterol (mmol/L)	1.70 (0.46)	1.67 (0.46)	1.59 (0.43)*
Systolic blood pressure (mm Hg)	129.0 (17.7)	133.2 (18.3)	136.7 (18.3)*
Diastolic blood pressure (mm Hg)	78.6 (10.6)	80.6 (11.0)	81.9 (10.9)*
HbA_1c_ concentration (%)	5.38 (0.54)	5.46 (0.63)	5.53 (0.65)*
Clustered metabolic risk score	−0.19 (0.59)	−0.04 (0.61)	0.13 (0.60)*
Physical activity (MET*hrs/day)	18.0 (7.1)	17.2 (6.8)	15.6 (6.7)*

*P<0.001;

†*P*<0.05;

‡*P*<0.01 across television viewing tertiles;

CVD: cardiovascular disease.

### Cox proportional hazards regression

**Figure 1:**
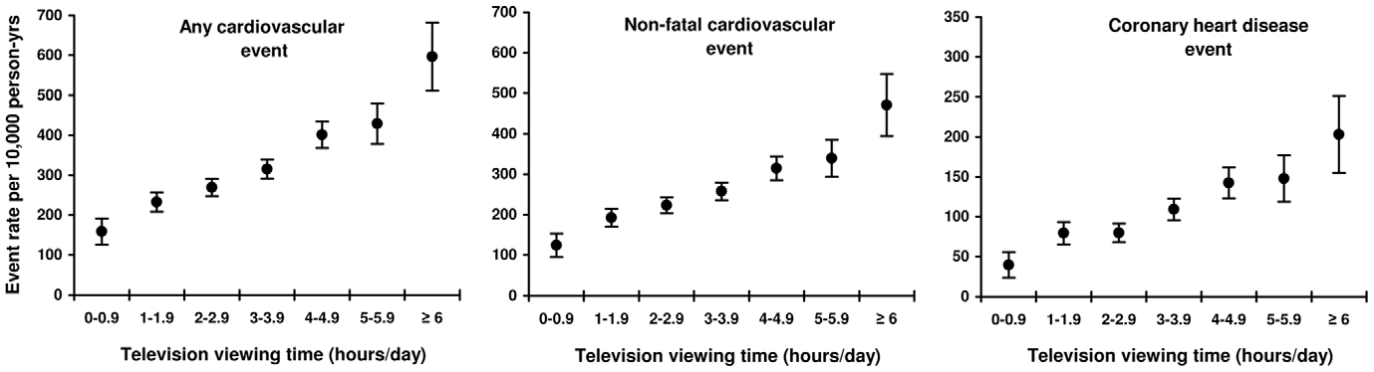
Age-adjusted cardiovascular disease event rates per 10,000 person-years by 1-hour increments of television viewing (hours/day). N television categories: 0–0.99: 800; 1–1.99: 2,126; 2–2.99: 3,180; 3–3.99: 3,057; 4–4.99: 2,040; 5–5.99: 926; ≥6: 479.

Figure 1 shows the increases in age-adjusted CVD event rates per 10,000 person-years by 1-hour increments in television viewing, suggesting a linear association between television viewing time and all 3 outcomes. Adding the quadratic television viewing term to Model C did not result in a significant improvement in model fit for any of the 3 outcomes.

As shown in Table 3, television viewing (hours/day) was positively associated with incident total CVD, non-fatal CVD and coronary heart disease, independent of age and gender (Model A), education, smoking, alcohol, hypertension, dyslipidaemia and antidepressant medication, baseline diabetes status, family history of CVD, sleep duration (Model B), and total PAEE (Model C). The increase in hazard associated with every one hour increase in television viewing time was 6% for non-fatal and total CVD, and 8% for coronary heart disease. These results were virtually unchanged after excluding participants (141 men and 96 women) who encountered any CVD event within the first year of follow-up (data not shown). Adjustment for social class instead of education level did not change results (data not shown).

**Table 3:** Hazard ratios (95%CI) for any cardiovascular, non-fatal cardiovascular and coronary heart disease events per hour/day increase in television viewing in 12,608 men and women in EPIC Norfolk, 1998–2007.

Model	Any cardiovascular event		Non-fatal cardiovascular event		Coronary heart disease event	
Models	Hazard ratio (95%CI)	*P*-value	Hazard ratio (95%CI)	*P*-value	Hazard ratio (95%CI)	*P*-value
Model A	1.10 (1.07–1.13)	<0.001	1.10 (1.07–1.14)	<0.001	1.14 (1.09–1.19)	<0.001
Model B	1.06 (1.03–1.09)	<0.001	1.07 (1.03–1.10)	<0.001	1.09 (1.04–1.14)	<0.001
Model C	1.06 (1.03–1.08)	<0.001	1.06 (1.03–1.09)	<0.001	1.08 (1.03–1.13)	<0.001

Participants with self-reported or diagnosed history of stroke, myocardial infarction or cancer at baseline were excluded.

Model A: adjusted for age and gender.

Model B: Model A additionally adjusted for education level, smoking status, alcohol consumption, medication for hypertension, medication for dyslipidaemia, medication for depression, baseline diabetes status, family history of cardiovascular disease and sleep duration.

Model C: Model B additionally adjusted for total physical activity energy expenditure (MET*hours/day).

Examination of the Schoenfeld residuals and the Kaplan-Meier plots indicated that the proportional hazards assumption was reasonable for these data.

After introducing multiplicative interaction terms to Model C, there were no significant interactions on any of the three outcomes between television viewing time and physical activity, BMI, gender, or education. A significant interaction by age and clustered metabolic risk was found for total CVD (age: *P* = 0.02; clustered metabolic risk: *P* = 0.03) and non-fatal CVD (age: *P* = 0.03; clustered metabolic risk: *P*<0.01), but not for coronary heart disease. Table 4 illustrates associations between television viewing and total CVD derived from Model C for subgroups. Consistent with the results of the interaction tests, very similar hazard ratios were found between subgroups for most characteristics. Older participants and participants with higher clustered metabolic risk respectively showed a somewhat lower hazard ratio for the association between television viewing time and incident total CVD compared to their younger and lower risk counterparts, although associations were still in the same direction.

**Table 4:** Hazard ratios (95%CI) for any cardiovascular disease event per hour/day increase in television viewing in subgroups according to physical activity level, body mass index, gender, education level, age and clustered metabolic risk, in 12,608 men and women in EPIC Norfolk, 1998–2007.

Classification by	Category (n° CVD events/n)	Hazard ratio (95%CI)
**PAEE level**	Low (1,663/6,109)	1.06 (1.02–1.09)
	High (957/6,499)	1.05 (1.01–1.10)
**Body mass index**	Normal weight (754/4,634)	1.03 (0.98–1.09)
	Overweight or obese (1,866/7,974)	1.06 (1.02–1.09)
**Gender** *	Men (1,351/5,465)	1.06 (1.02–1.10)
	Women (1,269/7,143)	1.05 (1.01–1.09)
**Education**	Low or O level (1,307/5,445)	1.06 (1.02–1.10)
	A level or Degree (1,313/7,163)	1.05 (1.01–1.09)
**Age**	≤60 year of age (591/6,106)	1.10 (1.04–1.16)
	>60 year of age (2,029/6,502)	1.04 (1.01–1.08)
**Clustered metabolic risk** †	Low (827/5,887)	1.08 (1.02–1.13)
	High (1,615/5,889)	1.03 (0.99–1.06)

Participants with self-reported or diagnosed history of stroke, myocardial infarction or cancer at baseline were excluded.

Models are adjusted for age, gender, education level, smoking status, alcohol consumption, medication for hypertension, medication for dyslipidaemia, medication for depression, baseline diabetes status, family history of cardiovascular disease, sleep duration and total physical activity energy expenditure.

*Models did not include gender;

†6.6% of participants had missing data.

Adding hormone replacement therapy use to model C in women resulted in the same hazard ratio for television viewing time (1.05 (1.01–1.09)), indicating that results in women were also independent of this factor.

As a second aim, potential mediation effects were examined by adding continuous energy intake, BMI, the clustered metabolic risk score and its individual metabolic risk variables to Model C. Preliminary analyses showed significant correlations between continuous television viewing time and these variables (all *P*<0.001; negative correlation with HDL-cholesterol, positive correlation with all other variables), and significant differences for these variables between incident cases and non-cases for the three outcomes (see Table 1), suggesting a mediation effect by these variables. As shown in Table 5, although an attenuation of the hazard ratios (95%CI) for television viewing was found in most models, they remained significant. This suggests that these characteristics only partially mediated the association between television viewing time and incident CVD. Additional inclusion of intake of fruit and vegetables (grams/day) and saturated fatty acids (% total energy intake) as indicators of an (un)healthy diet in the model examining a potential mediation effect of dietary intake (Model C+total energy intake) resulted in very similar hazard ratios (95%CI) for television viewing time: 1) total CVD: 1.07 (1.03–1.10), *P*<0.001; 2) non-fatal CVD: 1.07 (1.03–1.11), *P*<0.001; and 3) coronary heart disease: 1.09 (1.04–1.15), *P*<0.001.

**Table 5:** Hazard ratios (95%CI) for any cardiovascular, non-fatal cardiovascular and coronary heart disease events per hour/day increase in television viewing in 12,608 men and women in EPIC Norfolk, 1998–2007, after additional adjustment for potential mediating variables.

	Any cardiovascular event		Non-fatal cardiovascular event		Coronary heart disease event	
Potential mediating variable	Hazard ratio (95%CI)	*P*-value	Hazard ratio (95%CI)	*P*-value	Hazard ratio (95%CI)	*P*-value
Total energy intake*	1.07 (1.03–1.10)	<0.001	1.07 (1.03–1.11)	<0.001	1.10 (1.04–1.16)	<0.001
Body mass index	1.04 (1.02–1.07)	0.002	1.05 (1.02–1.08)	0.002	1.07 (1.02–1.12)	0.003
Waist circumference	104 (1.02–1.07)	0.003	1.05 (1.02–1.08)	0.002	1.07 (1.02–1.12)	0.003
Triglycerides*	1.05 (1.02–1.08)	0.002	1.05 (1.02–1.09)	0.001	1.07 (1.02–1.12)	0.004
HDL cholesterol*	1.04 (1.01–1.07)	0.004	1.05 (1.02–1.08)	0.003	1.06 (1.02–1.11)	0.010
Systolic blood pressure	1.05 (1.02–1.08)	<0.001	1.05 (1.02–1.09)	<0.001	1.08 (1.03–1.13)	<0.001
Diastolic blood pressure	1.05 (1.03–1.08)	<0.001	1.06 (1.03–1.09)	<0.001	1.08 (1.04–1.13)	<0.001
HbA_1c_ concentration*	1.05 (1.02–1.08)	<0.001	1.06 (1.03–1.09)	<0.001	1.08 (1.03–1.13)	0.002
Clustered metabolic risk*	1.04 (1.01–1.07)	0.016	1.04 (1.01–1.08)	0.010	1.06 (1.02–1.12)	0.009
Total energy intake+body mass index+clustered metabolic risk*	1.04 (1.01–1.08)	0.011	1.05 (1.01–1.09)	0.015	1.09 (1.03–1.15)	0.003

Participants with self-reported or diagnosed history of stroke, myocardial infarction or cancer at baseline were excluded.

Models are adjusted for age, gender, education level, smoking status, alcohol consumption, medication for hypertension, medication for dyslipidaemia, medication for depression, baseline diabetes status, family history of cardiovascular disease, sleep duration, total physical activity energy expenditure and the potential mediation variable as indicated.

*Variable had missing data: total energy intake: 20.9%, triglycerides: 5.8%, HDL cholesterol: 5.9%, HbA_1c_ concentration: 4.9%, clustered metabolic risk: 6.6%, total energy intake+clustered metabolic risk: 25.9%.

Additionally, we examined the association between television viewing time and incident cardiac failure (229 fatal or non-fatal events; men: 147, women: 82) and stroke (261 fatal or non-fatal events; men: 127, women: 134). Television viewing (hours/day) was positively associated with incident cardiac failure, independent of age and gender (Model A: HR (95%CI): 1.15 (1.05–1.25)), education, smoking, alcohol, hypertension, dyslipidaemia and antidepressant medication, baseline diabetes status, family history of CVD, sleep duration (Model B: HR (95%CI): 1.11 (1.01–1.21)), and total PAEE (Model C: HR (95%CI): 1.10 (1.01–1.20)). Positive but non-significant associations were found for incident stroke (HR (95%CI): Model A: 1.06 (0.98–1.16), Model B: 1.04 (0.95–1.13), Model C: 1.03 (0.95–1.12)).

## Discussion

In this population-based sample of healthy middle-aged white adults, higher television viewing time was independently associated with an increased hazard of incident total CVD, non-fatal CVD and coronary heart disease. These associations were independent of a continuous measure of overall (during leisure-time, to work, at work and at home) PAEE. Although PAEE was self-reported, this may suggest that these associations are not just the result of a compensatory reduction in physical activity. Furthermore, associations were equally strong in high and low physically active individuals and may support the need for recommendations on limiting television viewing time, in addition to the established physical activity recommendations [9]. So far, two studies reported independent associations between television viewing time and cardiovascular mortality, the final outcome in the CVD process [13], [14]. The recent findings from the Scottish Health Survey [16] indicated that leisure screen time (viewing television or another type of screen such as computer or video game) was associated with total (fatal and non-fatal combined) incident CVD (215 cases) in 4,512 adults (aged ≥35) followed up for 4.3 (±0.5) years. Despite minor differences in the exposure variable and adjustment strategy, the hazard ratio (95%CI (raised to power 60 to transform from minutes/day to hours/day)) for continuous screen time from the fully adjusted model for total incident CVD was very similar to the one in our study, more specifically 1.06 (1.01–1.11) [16]. Compared to the literature, the current study provides important novel findings by showing independent associations between television viewing time and not only non-fatal CVD, imposing a large burden on society, but also coronary heart disease. Additional analyses for incident cardiac failure and stroke suggested that the strength of association may differ to some extent according to the subtype of CVD outcome examined. Although results for incident cardiac failure and stroke should be interpreted with caution given the limited number of incident cases for both outcomes, these novel findings should encourage future larger prospective studies with longer follow up time to further explore these independent associations for specific types of CVD.

Every hour increase (or decrease) in television viewing time was independently associated with a 6% higher (lower) hazard for total and non-fatal CVD and an 8% higher (lower) hazard for coronary heart disease. Although this effect size is relatively modest, television viewing is a highly prevalent behaviour, both at intra- (e.g. on average nearly three hours/day in Great Britain) and inter-individual levels (e.g. reported by 80% of Great Britain adults) [19]. It therefore provides an important target for health-related behaviour change and cardiovascular health benefits at a population level.

Examining pathways explaining independent effects from television viewing time and (prolonged) sitting per se on cardiovascular health is a relatively unexplored area. A unique and important finding of this study is that a large range of intermediate behavioural and biological characteristics only partially mediated the observed associations. Effect attenuation by these characteristics is supported by results from previous studies. These include independent associations of television viewing time and objectively measured sedentary behaviour with increased energy intake [23], weight gain/incident obesity [2]–[4], dyslipidaemia [5], hypertension [6] and insulin resistance/diabetes [3], [7], [8], which are independent of physical activity and other relevant covariates; partial mediation by triglycerides and HDL cholesterol is additionally supported by findings in rat-models showing acute effects on skeletal muscle lipoprotein lipase which are, both quantitatively and qualitatively, very specific to enforced muscle inactivity conditions (simulating human sedentary behaviour) and largely uninfluenced by exercise conditions [9]. However, none of all potential mediators in the current study could completely account for the associations between television viewing and the outcomes. Further research is required on the (pathophysiological) processes which are specific for sedentary behaviours and not just the effect of absence of physical activity. As television viewing time and these intermediate characteristics were measured at the same time point, we cannot however disentangle direction of causality and therefore true mediation by these variables. Future observational and experimental studies with multiple time-points are warranted to further disentangle these pathways.

An important strength of this study is the large population-based sample of men and women, in which a relatively large proportion developed incident CVD. This enabled us to study three different types of CVD which have not been associated with television viewing time before, and also preliminary examine associations for incident cardiac failure and stroke. Given the sample size, we were able to exclude everyone with baseline history of self-reported and/or diagnosed stroke, myocardial infarction and cancer, and in sensitivity analysis those who developed CVD within the first year of follow-up. This, together with the prospective design and extensive adjustment for confounders, supports a causal inference of the associations found and reduces the possibility of reverse causality. We adjusted for overall PAEE (four different domains), a measure which has previously been shown to predict cardiovascular mortality in this cohort [27], in addition to demonstrated criterion-validity [26]. Furthermore, a comprehensive set of potential mediators were considered, including energy intake and indicators of diet quality, a wide spectrum of objectively measured cardiovascular risk factors and a summary cardio-metabolic risk score. A potential weakness that merits discussion is that television viewing time was self-reported, and only measured at baseline. Therefore, measurement error and misclassification might have resulted in an underestimation of the true associations. Further, although a large range of potential confounders was considered, residual confounding might exist for those that relied on self-report or were categorical. Additionally, although television viewing is the most-prevalent leisure time behaviour [19]–[21] and likely to be representative of leisure time sedentary behaviour [22], other types of sedentary behaviour correlating with television viewing time might also have contributed to residual confounding. Based on recent findings in American adults, showing significant but fair associations between TV viewing time and total sedentary time derived from accelerometry, TV viewing time has been suggested to be a potentially useful indicator for total sedentary time in epidemiological analyses. Of particular relevance to our population sample, rank order correlations in mid- and old-aged adults (Spearman's rho: 40–59 years olds: 0.17 (P<0.001); 60+ year olds: 0.23 (P<0.001) were stronger compared to those in younger adults (20–39 years old: 0.05 (P = 0.04)) [31]. As information on types of video or TV programs watched (e.g. highly charged sports events versus other shows) was not available, we could not examine the possibility of differences in strength of association according to type of entertainment content. The specific mediation effect of eating while watching TV viewing was also not studied as this behaviour was not measured separately, but we did adjust for general dietary variables. Finally, as we excluded participants with baseline chronic health conditions to minimize the possibility of reverse causality and increase internal validity, the associations found are relative to this cohort of relatively healthy middle-aged white adults. Additionally, non-randomly missing data might have biased results, especially in models including dietary intake, which involved exclusion of relatively large proportions of participants. Further prospective large population-based studies in different settings, using repeated measurements, with longer duration of follow-up and preferably using objective measures of physical activity and sedentary time are needed to extend the present results and confirm dose-response effects in different populations.

To conclude, television viewing time, the predominant leisure activity in modern society [19]–[21], was associated with increased risk of total incident CVD, non-fatal CVD and coronary heart disease in healthy middle-aged white adults, independent of total level of physical activity and other relevant confounders. Furthermore, an extensive range of intermediate CVD risk factors only partially mediated these independent associations. These observations suggest the need to consider separate public health recommendations on reducing television viewing time.

## Materials and Methods

### Study participants

EPIC Norfolk is part of the 10-country collaborative European Prospective Investigation into Cancer and Nutrition (EPIC) study. A cohort of 25,633 residents of Norfolk (UK) aged 45–79 and recruited via general practitioners agreed to participate between 1993–1997. A detailed description of the study design and cohort characteristics has previously been published [24]. Participants were invited for a follow-up assessment between January 1998–October 2000. This follow-up also encompassed introduction of the EPIC physical activity questionnaire (EPAQ2), which is a more comprehensive physical activity instrument also including questions on television viewing as described in more detail below. This follow-up examination, including 15,784 (61.6%) attendees, is the baseline for the current analyses. Complete data for television viewing time, PAEE, education level, smoking status, alcohol consumption, medication use, baseline diabetes status, parental history of CVD and sleep duration were provided by 15,021 participants. Those with diagnosed or self-reported baseline history of stroke (n = 367), myocardial infarction (n = 635), other vascular disease (n = 3) and/or cancer (n = 1,449) were excluded. As a result, 12,608 participants (5,465 men, 7,143 women) were included in the current analyses. The study complies with the Declaration of Helsinki, and was approved by The Norwich District Health Authority Ethics Committee. All participants provided written informed consent.

### Measures

#### Incident CVD events

All EPIC Norfolk participants are followed up for fatal and non-fatal CVD events. The present study includes follow-up until 31^st^ March 2007, covering 6.9±1.9 (mean±SD) follow-up years. The Office of National Statistics, UK, flagged all individuals for death certification and trained nosologists coded death certificates according to the International Classification of Disease (ICD). Cardiovascular death was defined as ICD 410–448 (ICD 9) or ICD I10–I79 (ICD 10) as underlying cause of death, which comprizes coronary heart disease (410–414 (ICD 9) or I20–I25 (ICD 10)), stroke (430–438 (ICD 9) or I60–I69 (ICD 10)), cardiac failure (428 (ICD 9) or I50 (ICD 10)) and other vascular causes. Cause-specific (same ICD coding) hospital admission was determined via ENCORE (East Norfolk Commission Record, the hospital admissions database kept by the East Norfolk Health Commission) [25], using individuals' unique National Health Service number. Death certificates and ENCORE show high accuracy in correctly identifying incident disease, as previously shown in EPIC Norfolk for incident stroke [25].

The primary outcome was any first CVD event during follow-up, a combined end point defined as the first of any of these events: hospital admission or death because of coronary heart disease, stroke or other vascular disease. Those with non-fatal CVD events were identified as having been admitted to hospital for any of those reasons without dying during follow-up. The subgroup of individuals with coronary heart disease events only included those who had coronary heart disease as the underlying cause of hospital admission and/or death during follow-up.

#### Television viewing time and PAEE

By means of the EPAQ2, participants self-reported their physical (in)activity behaviour in different sub-domains (at home, to work, at work and during leisure-time), using the past year as a reference frame [26]. As previously described in detail [27], PAEE (MET*hours/week) was calculated in each of these four (mutually exclusive) sub-domains by multiplying participation (hours/week) by the metabolic cost of each activity (metabolic equivalent (MET)) according to Ainsworth et al. [28]. Total PAEE was then determined by summing energy expenditure of the different sub-domains. Time spent watching television and video (hours/week) was calculated based on responses to four questions about watching before and after six pm at week- and weekend-days. The EPAQ2 scored high for repeatability, both in terms of television viewing time and PAEE [26]. It is valid for ranking individuals, as shown by comparison against minute-by-minute heart rate monitoring and maximal aerobic capacity (VO_2max_) [26]. In the current study, television viewing time and PAEE were expressed in hours/day and MET*hours/day respectively, for ease of interpretation; they were mutually exclusive (i.e. television viewing was not part of PAEE).

#### Covariates

Participants self-reported their education level (low, O level, A level, degree), social class (manual, non manual), smoking status (current, former, never), alcohol consumption (units/week), medication for diabetes (yes, no), medication for hypertension (yes, no), medication for dyslipidaemia (yes, no), medication for depression (yes, no), hormone replacement therapy status (current, former, never), baseline history (yes, no) of myocardial infarction, stroke, cancer and diabetes, and family history of CVD (yes, no) by means of a detailed health and lifestyle questionnaire [24]. Sleep duration (hours/day) was assessed using the EPAQ2 [26]. Diagnosed history of cancer was identified by the ECRiC (Eastern Cancer Registry and Information Centre) and the Office of National Statistics. Total energy intake (kj/day), fruit and vegetable intake (g/day) and saturated fatty acids intake (% total energy intake) were derived from a validated 130-item semi-quantitative food frequency questionnaire [29].

As previously described in detail [24], trained nurses measured height, weight and waist circumference following standardized protocols. Body mass index (BMI) was calculated as weight divided by (height)^2^ (kg/m^2^). Systolic and diastolic blood pressures were measured in duplicate using an Accutorr sphygmomanometer (Datascore, UK) after three minutes of sitting. A non-fasting venous blood sample was examined for triglycerides and high density lipoprotein (HDL) cholesterol (mmol/L) using RA1000 (Bayer Diagnostics, UK). Glycated haemoglobin (HbA_1c_) was measured using Diamat ion exchange HPLC (Bio-Rad Laboratories, UK). A clustered metabolic risk score was constructed to summarize cardio-metabolic risk based on continuously distributed indicators of central obesity (waist circumference), dyslipidaemia (triglycerides and HDL cholesterol), hypertension (systolic and diastolic blood pressure), and hyperglycemia (HbA_1c_) [30]. These variables were standardized (i.e. z-scores were computed (z = ([value−mean]/SD)), after normalizing (log10) triglycerides and HbA_1c_. Systolic and diastolic blood pressure z-scores were averaged and the HDL z-scores were inverted. Z-scores were summed and the sum was divided by five to express the cardio-metabolic risk score in SD units. Standardization (z-scores) was performed, stratified by sex (i.e. in males, the male-specific mean and SD values were used for each metabolic variable, in females, the female-specific mean and SD values were used for each metabolic variable) including all participants with complete data for each metabolic variable.

### Statistical analysis

Analyses were conducted using SPSS15.0 (SPSS, Inc., Chicago, IL) and STATA10.0 (Stata, Corp., TX). Statistical significance was set at *P*<0.05. Baseline characteristics were compared by incident CVD status (unpaired t-tests and chi-squared tests) and by television viewing tertiles (lowest: <2.5, middle: 2.5–3.6, highest tertile: >3.6 hours/day; one-way ANOVA and chi-squared tests).

To examine the association between baseline television viewing time (hours/day) and incident CVD Cox proportional hazards regression was used. The proportional hazards assumption was checked by examining Schoenfeld residuals and Kaplan-Meier plots for all three outcome variables. The Schoenfeld residuals did not suggest evidence of deviations from proportionality, which was consistent with observations in the Kaplan-Meier plots. To examine linearity of the association between television viewing time and the outcomes, age-adjusted CVD event rates (95%CI) per 10,000 person-years of follow-up were plotted by 1-hour increments in television viewing time. Additionally, a log likelihood-ratio test examined whether addition of a quadratic term for television viewing time to the adjusted model (Model C) resulted in a statistically significant improvement in model fit. Based on evidence for a linear association, hazard ratios (95%CI) per hour/day increase in television viewing time were estimated. Initial models (Model A) were adjusted for baseline age and gender, and were then further adjusted (Model B) for baseline education level, smoking status, alcohol consumption, antihypertensive medication, lipid-lowering medication, antidepressant medication, diabetes status (based on diabetes history and diabetes medication use), family history of CVD and sleep duration. To examine whether television viewing time was independently associated with incident CVD, we then included baseline PAEE in the model (Model C). Analyses were repeated after additionally excluding participants who experienced any CVD event within the first year of follow-up, to examine the possibility that preclinical disease might influence television habit.

Multiplicative interaction terms were added to Model C to examine effect modification by physical activity level (median split: ≤15.7, >15.7 MET*hours/day), BMI (normal weight: <25, overweight/obese: ≥25 kg/m^2^), gender, education level (low/O level, A level/degree), age (≤60, >60 yrs of age) and clustered metabolic risk (median split: ≤−0.05, >−0.05). As 6.6% of participants had missing data for clustered metabolic risk, this specific model included 11,776 participants.

The following potential mediators of the association between television viewing time and incident CVD were examined by introducing them into Model C: total energy intake (kJ/day; 21% missing; with and without additional inclusion of fruit and vegetable intake (g/day) and saturated fatty acids intake (21% missing)); BMI, waist circumference, triglycerides (5.8% missing), HDL-cholesterol (5.9% missing), systolic and diastolic blood pressure, HbA_1c_ (4.9% missing) and the clustered metabolic risk score (6.6% missing). Finally, total energy intake, BMI and the clustered metabolic risk score were entered simultaneously (25.9% missing) to Model C. Due to additional missing data for some of these potential mediators, the corresponding models did not include all 12,608 participants.
